# Estimation of Activity Interaction Parameters in Fe-S-j Systems

**DOI:** 10.3390/e20100808

**Published:** 2018-10-22

**Authors:** Tianhua Ju, Xueyong Ding, Yingyi Zhang, Weiliang Chen, Xiangkui Cheng, Bo Wang, Jingxin Dai, Xinlin Yan

**Affiliations:** 1 School of Metallurgy, Northeastern University, Shenyang 110004, China; 2 School of Metallurgical Engineering, Anhui University of Technology, Maanshan 243002, China; 3 Institute of International Vanadium Titanium, Panzhihua University, Panzhihua 617000, China; 4 Institute of Solid State Physics, Vienna University of Technology, Wiedner Hauptstr. 8-10, 1040 Vienna, Austria

**Keywords:** interaction parameter model, miedema model, iron-based melts, desulphurization thermodynamics

## Abstract

It is important to know the activity interaction parameters between components in melts in the process of metallurgy. However, it’s considerably difficult to measure them experimentally, relying still to a large extent on theoretical calculations. In this paper, the first-order activity interaction parameter ($e_{s}^{j}$) of ***j*** on sulphur in Fe-based melts at 1873 K is investigated by a calculation model established by combining the Miedema model and Toop-Hillert geometric model as well as considering excess entropy and mixing enthalpy. We consider two strategies, with or without using excess entropy in the calculations. Our results show that: (1) the predicted values are in good agreement with those recommended by Japan Society for Promotion of Science (JSPS); and (2) the agreement is even better when excess entropy is considered in the calculations. In addition, the deviations of our theoretical results from experimental values $\left|e_{S\left(\exp\right)}^{j}-e_{S\left(\mathrm{cal}\right)}^{j}\right|$ depend on the element ***j***’s locations in the periodic table.

## 1. Introduction

Sulphur is one of the most detrimental impurity elements in metallurgy that typically causes the deterioration of hot ductility [1] and the degradation of the corrosion resistance [2] of steels. The content of sulphur in steels is normally required to be extremely low. “Inclusion engineering” [3] could be one of the ways to reduce the harmful effects of sulphur [4] with a relatively low cost. However, implementation of this technique needs to well understand the basic thermodynamics behavior of sulphur in iron-based melts.

The activity interaction parameter, which is first introduced by Wagner [5] in dilute solution to account for the effects of an added alloying element on the activity coefficient of a solute, provides more useful information in the process of metallurgy computation. Previously, only first-order activity interaction parameters had been considered in Wagner’s formalism, resulting in inadequacy to describe the behavior of solutions that are “not very diluted”. This phenomenon was observed by Lupis and Elliott [6], who then proposed an introduction of higher order interaction coefficients to the mathematical apparatus. Darken [7] also observed that the Wagner’s formalism was not suited to the non-dilute solution situationnand suggested a quadratic formalism by considering the first- and second-order activity interaction parameters. Pelton and Bale [8,9] further developed Darken’s quadratic formalism by introducing the unified interaction parameter (UIP) to the formalism. In UIP, the first-order interaction parameter is identical to Wagner’s first-order interaction parameter. Therefore, the activity interaction parameter is an extremely important and fundamental thermodynamics parameter in the fields of metallurgy and materials. In addition, the activity interaction parameters also bear significant correlations among properties such as the heat of formation of the corresponding oxides and atomic number of the deoxidants [10], as well as how the solubility of one element in a liquid metal is affected by the second solute [11]. Therefore, knowing the activity interaction parameter in the Fe-S-j systems is very important to understand the thermodynamic behavior of sulphur in steel.

The activity interaction parameter can be basically determined by experimental methods. However, it is practically impossible to determine all these parameters due to the large number of potential elements for combining systems and possible technical issues. As a result, theoretical methods become most attractive approaches. In theoretical works, two methods are deserved to be mentioned since in which, only few physical parameters of constituent elements are needed. One is proposed by Ding [12,13] and the other is developed by Ueno and Waseda [14]. Ding [12,13] at the early 1990s proposed a method that, through combing the Miedema model and geometric model as well as including other thermodynamics relations, established a model to predict the activity interaction parameter and infinite dilute activity coefficient in any metal-based melt. Almost at the same time, Ueno and Waseda [14] applied the pseudopotential formalism coupled with the free energy of a hard sphere model and built a model for the activity interaction parameter in metal-based melts. The former we called as Ding method and the latter as Ueno method. In the Ueno method, the final solution formula needs to improve because it does not satisfy the Lupis reciprocal relationship [6], i.e., This problem does not exist in the Ding method.

After the Ding method, many prediction models have been established based on it. For example, Fan [15] coupled Chou’s geometric solution model with the Miedema model, Wang [16] applied Toop’s geometric model as extending method, Zhang [17] combined the Miedema model and Chou’s geometric solution model and also included excess entropy, etc. The prediction capability of the Ding method totally relies on the Miedema model. In the past, Ding coupled the Miedema model with Toop-Kholer geometric model to calculate the activity interaction parameter of solutes in Fe-based [12,18], Cu-based [18], and Co-based [18] melts, respectively, and the predicted data are in good agreement with the experimental data. In these calculations, however, data on sulphur with other solutes are not included due to the fact the physical parameters (given by Miedema et al.) of sulphur which were given by Neuhausen [19] were not available until 2003. Thence, applying the Ding method to calculate the activity interaction parameter of sulphur with other solutes has become possible.

In iron-based melts, due to the importance of sulphur for the properties of steel, many activity interaction parameters of sulphur with other solutes have been determined experimentally. The results are compiled in “*Thermodynamic Data for Steelmaking*” [20] edited by the Japan Society for the Promotion of Science (JSPS). However, data on the activity interaction parameters of sulphur with some important elements such as Rh, Ru, Er, Os, Re, etc. are still missing. In addition, the experimental data are usually inconsistent from different sources. For example, the $e_{O}^{Ca}$ given by Inoue et al [21] is −9000, however, the value given by JSPS [20] is −515. Therefore, applying the theoretical method to predict the activity interaction parameter in Fe-S-***j*** has practical significance.

In this work, the activity interaction parameters (in which the composition coordination is expressed in mass%) in Fe-based melts were calculated by establishing a model based on the Ding method. Although many models based on the Ding’s method for activity interaction parameter calculations have been established, most of them have problems in use. The models coupled with Chou’s model [15,17], for instance, have the problem that the similarity coefficient is difficult to obtain. The models combined with the Toop/Toop-Kholer geometric model, such as Ding’s model [12] and Wang’s model [16], have mathematical difficulties in the deduction process when the solvent is chosen as an asymmetric component and one has to resort to other geometric models. For this reason, in our present work, we adopted the Toop-Hillert geometric model [22] in our model establishment.

## 2. Calculation Method

### 2.1. Basic Relations

In a ternary system, ***i-j-k***, ***k*** is a solvent, the activity interaction parameter $\varepsilon_{i}^{j}$ can be expressed as:

(1) $$\varepsilon_{i}^{j}=\frac{1}{RT}{\left(\frac{\partial^{2}g^{E}}{\partial x_{i}\partial x_{j}}\right)}_{x_{k}\to 1}$$

and:

(2) $$g^{E}=\Delta H-TS^{E}$$

where *R* and *T* are the gas constant and absolute temperature, respectively; is the activity interaction parameter of ***j*** on ***i*** that the composition coordinate is in a molar fraction; $g^{E}$ and $S^{E}$ are the excess Gibbs free energy and excess entropy, respectively; Δ*H* is the mixing enthalpy of solution.

Generally, the thermodynamics properties of a multi-component system are obtained from all the sub-binary systems with an assigned probability weights, which is called geometric model method, as follows:

(3) $$g^{E}=\sum_{i}\sum_{j=i+1}w_{ij}g_{ij}^{E}$$

Therefore, when the excess Gibbs free energy of the binaries is available, the excess Gibbs free energy of the ***i-j-k*** system, *g^E^*, can be obtained, and then the activity interaction parameter $\varepsilon_{i}^{j}$ can be calculated.

In liquid binary alloys, a satisfactory equation relating *S^E^* and Δ*H* has been deduced by Tanaka et al. [23], based on the free volume theory and excess volumes of the alloys, as follows (supposing and ***i-j*** binary alloy):

(4) $$S_{\mathrm{ij}}^{E}=\frac{1}{14}\Delta H_{ij}\left[1/T_{\mathrm{mi}}+1/T_{\mathrm{mj}}\right]$$

where $T_{mi}$ and $T_{mj}$ are the melting points of pure elements A and B under the standard pressure respectively. Therefore, if:

(5) $$\alpha_{ij}=\left[1-\frac{1}{14}T\left(1/T_{\mathrm{mi}}+1/T_{\mathrm{mj}}\right)\right]$$

then:

(6) $$g_{ij}^{E}=\alpha_{ij}\Delta H_{ij}$$

### 2.2. Miedema Model

In a binary system ***i-j***, the mixing enthalpy Δ*H_y_* can be obtained from the Miedema model [24,25,26], which was proposed by Miedema and his colleagues for estimating the heat of formation of solid or liquid metal alloys. For simplicity, the equation is deduced as follows:

(7) $$\Delta H_{ij}=f_{ij}\frac{x_{i}V_{i}^{2/3}\left[1+\mu_{i}x_{j}\left(\phi_{i}-\phi_{j}\right)\right]x_{j}V_{j}^{2/3}\left[1+\mu_{j}x_{i}\left(\phi_{j}-\phi_{i}\right)\right]}{x_{i}V_{i}^{2/3}\left[1+\mu_{i}x_{j}\left(\phi_{i}-\phi_{j}\right)\right]+x_{j}V_{j}^{2/3}\left[1+\mu_{j}x_{i}\left(\phi_{j}-\phi_{i}\right)\right]}$$

where:

$$f_{ij}=\frac{2p}{{\left(n_{ws}^{1/3}\right)}_{i}^{-1}+{\left(n_{ws}^{1/3}\right)}_{j}^{-1}}\times \left[\frac{q}{p}{\left({\left(n_{ws}^{1/3}\right)}_{i}-{\left(n_{ws}^{1/3}\right)}_{j}\right)}^{2}-{\left(\phi_{i}-\phi_{j}\right)}^{2}-0.73\beta_{ij}\right],$$

where, x is the atom fraction; *V*, $n_{ws}$, and ϕ are the basic physical parameters of elements, representing mole volume, electron density, and electronegativity, respectively; *p*, *q*, and *β_ij_* are the empirical parameters defined by Miedema, and their data are correlate to the constituents. The values of molar volume, electron density, and electronegativity of elements, except for O, S, Se, Te, as well as values of all the constants, are obtainable in reference [26].

### 2.3. Hillert-Toop Geometric Model

The Toop-Hillert geometric model [22] is used in this work to represent the excess Gibbs free energy of a ternary system, ***i-j-k***, from the three sub-binaries ***i-j***, ***i-k***, ***j-k***. The Toop-Hillert geometric model is an asymmetric model. Hence, the exact expression depends on the selected asymmetric component. If the component i is an asymmetric component, the excess Gibbs free energy *g^E^* can be expressed as:

(8) $$\begin{array}{l} g^{E}=\frac{x_{j}}{1-x_{i}}g_{ij}^{E}(x_{i};1-x_{i})+\frac{x_{k}}{1-x_{i}}g_{ik}^{E}(x_{i};1-x_{i})+\frac{x_{k}}{1-x_{j}}g_{jk}^{E}(x_{j};1-x_{j})+\\ \;\;\frac{x_{j}}{1-x_{k}}g_{kj}^{E}(x_{k};1-x_{k}) \end{array}$$

### 2.4. Calculation Model

Inserting Equations (5) and (7) into Equation (6), then expanding the formalism as Equation (8) to calculate the excess Gibbs free energy in a ternary system, finally, according to the Equation (1), the formalism for activity interaction parameter calculation is obtained:

1. If the asymmetric component is solute, the activity interaction parameter can be calculated by:
  (9) $$\varepsilon_{i}^{j}=\frac{1}{RT}\left[A-C-D+G\right]$$
2. If the solvent is an asymmetric component, the activity interaction parameter is:
  (10) $$\varepsilon_{i}^{j}=\frac{1}{RT}\left[A+B+E+F\right]$$
  where
  $A=f_{ij}^{*}\left[1+\mu_{i}\left(\phi_{i}^{}-\phi_{j}^{}\right)\right]/V_{j}^{2/3}$
  $B=f_{ij}^{*}\left[1+\mu_{i}\left(\phi_{j}^{}-\phi_{i}^{}\right)\right]/V_{i}^{2/3}$
  $C=f_{ik}^{*}\left[1+\mu_{i}(\phi_{i}-\phi_{k})\right]/V_{k}^{2/3}$
  $D=f_{jk}^{*}\left[1+\mu_{j}(\phi_{j}-\phi_{k})\right]/V_{k}^{2/3}$
  $\begin{array}{l} E=f_{jk}^{*}\{\left[[1-\mu_{j}(\phi_{j}-\phi_{k})]\left[V_{j}^{2/3}(1+\mu_{j}(\phi_{j}-\phi_{k}))+V_{k}^{2/3}(-1+\mu_{k}(\phi_{k}-\phi_{j}))\right]/{\left(V_{k}^{2/3}\right)}^{2}\right]\\ \;\;+\left[(2\mu_{j}+\mu_{k})(\phi_{k}-\phi_{j})-1-\mu_{j}\mu_{k}{\left(\phi_{k}-\phi_{j}\right)}^{2}\right]/V_{k}^{2/3}\} \end{array}$
  $\begin{array}{l} F=f_{ik}^{*}\{[(2\mu_{i}+\mu_{k})(\phi_{k}-\phi_{i})-1-\mu_{i}\mu_{k}{(\phi_{k}-\phi_{i})}^{2}]/V_{k}^{2/3}-[1+\mu_{i}\left(\phi_{i}-\phi_{k}\right)]\\ \;\;[V_{i}^{2/3}(1+\mu_{i}\left(\phi_{i}-\phi_{k}\right))+V_{k}^{2/3}\left(-1+\mu_{k}\left(\phi_{k}-\phi_{i}\right)\right)]/{\left(V_{k}^{2/3}\right)}^{2}\} \end{array}$
  $\begin{array}{l} G=f_{jk}^{*}\{[(2\mu_{j}+\mu_{k})(\phi_{k}-\phi_{j})-1-\mu_{j}\mu_{k}{\left(\phi_{k}-\phi_{j}\right)}^{2}]/V_{k}^{2/3}-\left[1+\mu_{j}(\phi_{j}-\phi_{k})\right]\\ \;\;\left[V_{j}^{2/3}(1+\mu_{j}(\phi_{j}-\phi_{k}))+V_{k}^{2/3}(-1+\mu_{k}(\phi_{k}-\phi_{j}))\right]/{\left(V_{k}^{2/3}\right)}^{2}\} \end{array}$
  here:
  $$f_{ij}^{*}=\frac{2pV_{j}^{2/3}V_{i}^{2/3}}{{\left(n_{ws}^{1/3}\right)}_{i}^{-1}+{\left(n_{ws}^{1/3}\right)}_{j}^{-1}}\left[\frac{q}{p}{\left(\Delta n_{WS}^{1/3}\right)}_{ij}^{2}-{\left(\Delta \phi^{*}\right)}_{ij}^{2}-a{\left(\frac{r}{p}\right)}_{ij}\right]*\alpha_{ij}$$
  where the *α_ij_* is identical to Equation (5).

If the mass fraction (wt.%) is used, the activity interaction parameter is often denoted as $e_{i}^{j}$, and it can be obtained by applying the below transformation from $\varepsilon_{i}^{j}:$

(11) $$e_{i}^{j}=(\varepsilon_{i}^{j}-(M_{k}-M_{j})/M_{k})\cdot \frac{M_{k}}{M_{j}}\cdot \frac{1}{230}$$

where $M_{j}$ and $M_{k}$ are the molecular weight of solute j and solvent k, respectively. In this paper, $e_{i}^{j}$ is used.

## 3. Results and Discussion

The Miedema parameters [19] of O, S, Se, Te, and Po required in the calculation of $e_{i}^{j}$ are listed in Table 1. The rule for selecting the asymmetric component is according to the criterion described in [27]. The temperature for the calculations of $e_{i}^{j}$ is 1873 K, and calculations are performed with (*S^E^* ≠ 0, case 1) or without considering (*S^E^* = 0, case 2) the excess entropy. When *S^E^* = 0, the *α_ij_* is equal to 1, else it is identical to Equation (5). The calculated results and experimental values recommended by JSPS [20] are listed in Table 2.

From Table 2, it’s noted that the calculated results are very encouraging. Over 85 percent of the predictions has the correct sign of $e_{S}^{j}$ compared to the experimental data in both cases. In the case 1, there are five data inconsistent in sign, in the case 2, there are only four. The absolute values are in general reasonable, except that an especially strong interaction exists between the S and ***j***, where the absolute values are smaller than the experimental data recommended by JSPS [20], as shown for $e_{S}^{Ca}$, $e_{S}^{La}$, $e_{S}^{Ce}$ in Table 2. However, due to the experimental difficulties at high-temperatures, experimental values from different labs for some components are scattered. For example, $e_{S}^{Ca}$ measured by Taguchi et al. [28] is −22.4 ± 6.4, but by Inoue et al. [21] it is −269 ± 28, respectively; the $e_{S}^{La}$ and $e_{S}^{Ce}$ obtained by Wu et al. [29] are −1.56 and −2.11, and given by JSPS are −18.3 and −9.1, respectively.

**Figure 1:**
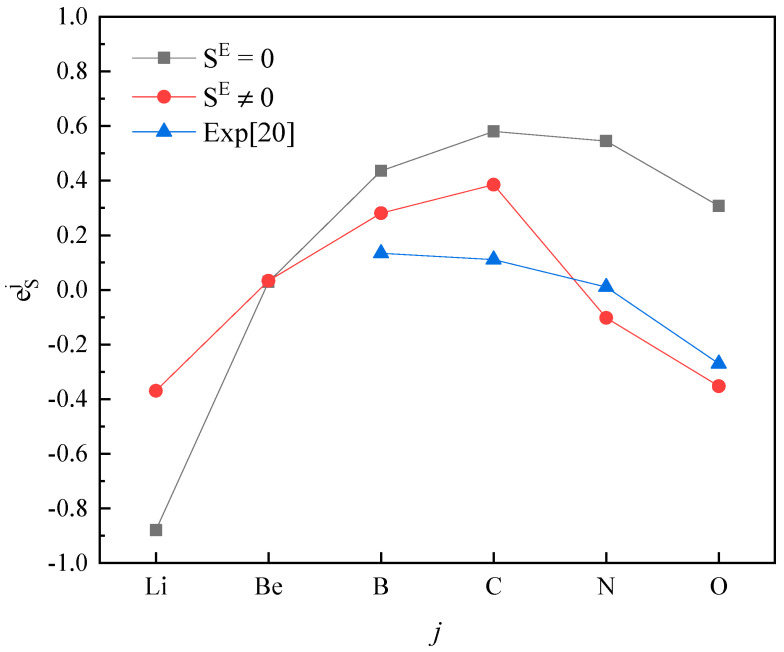
The activity interaction parameter of the 2nd periodic elements ***j*** on S.

**Figure 2:**
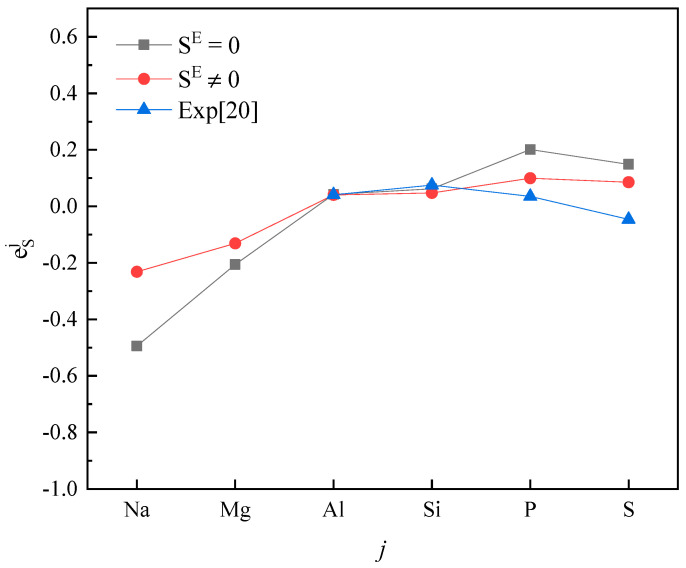
The activity interaction parameter of the 3rd periodic elements ***j*** on S.

**Figure 3:**
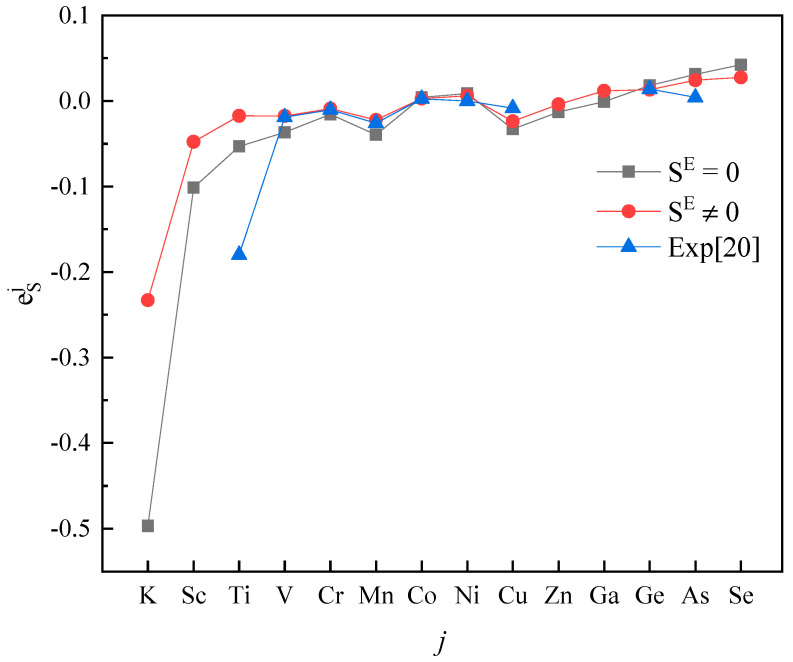
The activity interaction parameter of the 4th periodic elements ***j*** on S.

**Figure 4:**
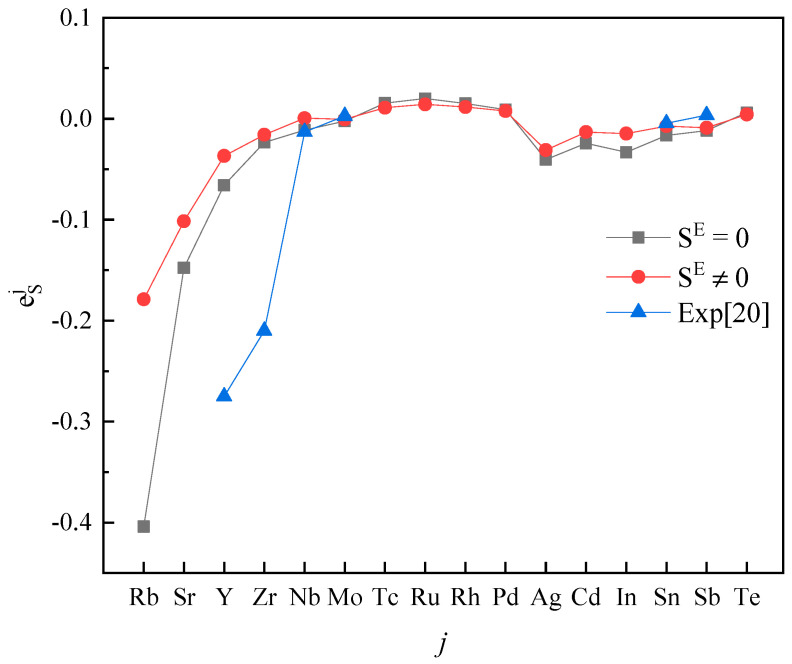
The activity interaction parameter of the 5th periodic elements ***j*** on S.

**Figure 5:**
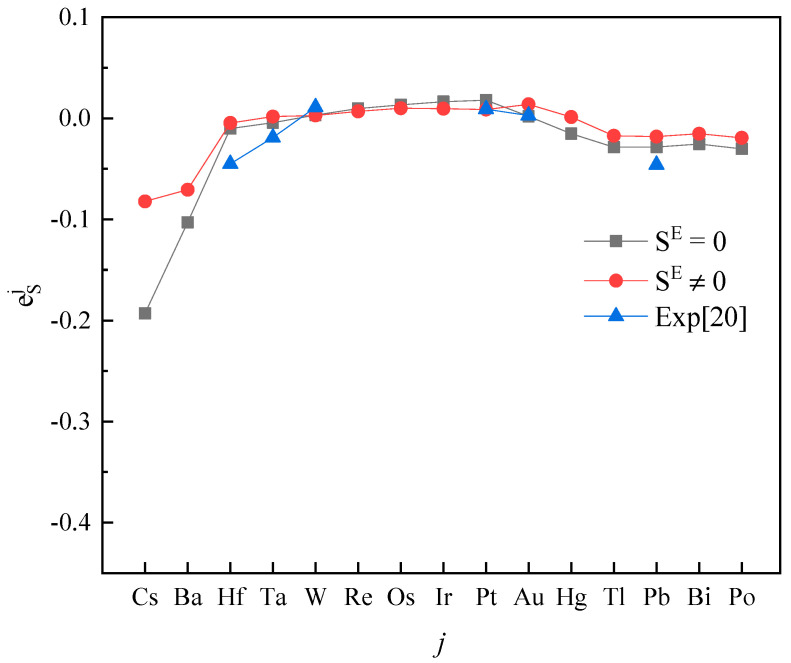
The activity interaction parameter of the 6th periodic elements ***j*** on S.

Plotting calculated values (for $S^{E}=0$ and $S^{E}\neq 0$) and experimental values according to incremental order of elements in each period of the periodic table, one can see the same trends among them (Figure 1, Figure 2, Figure 3, Figure 4 and Figure 5). Our results, however, differ from those by Silva [10], who showed the experimental $e_{O}^{i}$ ($\varepsilon_{O}^{j}$) increases linearly with increasing atomic number. In addition, it’s obvious (Figure 1, Figure 2, Figure 3, Figure 4 and Figure 5) that a better agreement is achieved when $S^{E}\neq 0$ (case 1) between theoretical values and experimental ones. Thus, theoretically, considering the excess entropy is more favorable.

**Figure 6:**
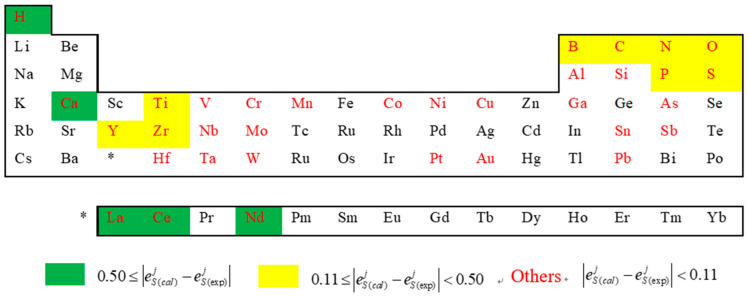
The distribution locations of elements ***j*** in periodic table together with the relative deviations of the corresponding activity interaction parameter $e_{S}^{j}$ between calculated and experimental values (the elements with red color represent that the experimental value of $e_{S}^{j}$ is available, the others represent that the experimental value is not available).

To establish our calculation method, we noticed that the precision of the calculated results heavily relies on the Miedema model and the geometric model. The Miedema model is one of the most successful models to predict the formation enthalpy of alloys. It may be owing to that more physical quantities such as electronegativity, electron density, and molar volume than other models like the Pauling electronegativity model (only electronegativity considered) have been considered [24]. However, it’s not sufficient when the constituents’ physical properties are of large differences, some minor contribution terms which are usually neglected now can’t be ignored. In the Fe-S-***j*** system, $e_{S}^{j}$ is not only dependent on the physical property differences between sulphur and elements ***j***, but also on the differences between elements ***j*** and iron as well as between iron and sulphur. Consequence, the elements *j* with large deviations between calculated and experimental values $\left|e_{S\left(\exp\right)}^{j}-e_{S\left(\mathrm{cal}\right)}^{j}\right|$ are mainly located in the periodic table far from the Fe group, especially in the left side (Figure 6), for example, the elements Ca, Ce, La, Y, Zr, etc. Moreover, the elements H, B, C, N, P, etc. are dealt with in Miedema’s model in a complicated way, and in current model, this may be also need some appropriate corrections to be made. Aiming at the deviation of the Miedema’s model, it can be improved by adding some terms such as a volume correction term [30] and an improved atomic size term [31], as well as by modifying the Miedema parameters of a specified element [32].

In addition, the energy of triplet interactions is neglected in our calculation model due to the contribution of this term to the excess Gibbs free energy *g^E^* of ternary elements is usually very small [33]. To see the influence of the geometric model, the results from the Ding’s model [12], which also includes a geometric model, are listed in Table 2 for comparison. One can see large deviations from experiments in both the present and Ding’s models. This means the deviations come mainly from the Miedema model basis instead of the geometric model. We are attempting to modify the Miedema model by adding some terms such as a volume correction term [30] and/or improving the atomic size factor [31] to optimize the calculated values. This work is now ongoing.

## 4. Conclusions

Because the activity interaction parameter $e_{S}^{j}$ is very important to understand the thermodynamic properties of Sulphur-contained iron-based melts, a great deal of work has been done on the experimental measurements. However, important data such as $e_{S}^{Ru}$, eSRe, $e_{S}^{Os}$, etc. are still lacking. Considering the complexity of measurements and the experimental data depend strongly on the experimental techniques, in this work we employed a theoretical method and systematically calculated the activity interaction parameter $e_{S}^{j}$ in the Fe-S-j systems. Based on our study, we conclude:

(1) A model for calculating the activity interaction parameter in a ternary system was established based on the Ding’s method, wherein the Toop-Hillert model was used.

(2) The calculated results for $e_{S}^{j}$ in Fe-based melts (Fe-S-*j*) by current model at 1873 K, with or without considering the excess entropy, show that better results would be obtained with considering the excess entropy. And better results would be obtained for the elements *j* located in the middle of periodic table nearby the Fe group.

(3) The reason for the large deviations between calculated and experimental values is because of the inaccuracy of Miedema’s model when the constituents’ physical properties are of large differences.

## Figures and Tables

**Table 1:** Miedema parameters [19] of the elements O, S, Se, Te, and Po.

Element	ϕ	$V^{2/3}$	$n_{WS}^{1/3}$	*μ*
O	6.97	2.66	1.70	0.04
S	5.60	4.38	1.46	0.04
Se	5.17	5.17	1.40	0.04
Te	4.72	6.44	1.31	0.04
Po	4.44	7.04	1.15	0.04

**Table 2:** Comparison of the calculation activity interaction parameter $e_{S}^{j}$ with experimental values recommended by JSPS [20] in Fe-based alloys at 1873 K.

j	Current Model		Ding Model [12]	JSPS	j	Current Model		Ding Model [12]	JSPS
	$S^{E}=0$	$S^{E}\neq 0$				$S^{E}=0$	$S^{E}\neq 0$		
H	4.0396	4.5582	1.3551	0.41	Pd	0.0089	0.0077	−0.0192	
Li	−0.8793	−0.3694	−0.7415		Ag	−0.0403	−0.0310	−0.0107	
Be	0.0297	0.0334	−0.0046		Cd	−0.0242	−0.0131	−0.0138	
B	0.4360	0.2808	0.3909	0.134	In	−0.0332	−0.0147	−0.0044	
C	0.5800	0.3849	0.3770	0.111	Sn	−0.0166	−0.0072	0.0009	−0.0044
N	0.5449	−0.1025	0.3709	0.01	Sb	−0.0117	−0.0090	0.0101	0.0037
O	0.3075	−0.3524	0.3574	−0.27	Te	0.0062	0.0042	−0.0738	
Na	−0.4944	−0.2318	−0.2903		Cs	−0.1929	−0.0822	−0.0567	
Mg	−0.2058	−0.1314	−0.1475		Ba	−0.1029	−0.0707	−0.0222	
Al	0.0426	0.0405	0.0112	0.041	Hf	−0.0103	−0.0047	−0.0116	−0.045
Si	0.0621	0.0471	0.0941	0.075	Ta	−0.0044	0.0016	0.0032	−0.019
P	0.2013	0.0995	0.1907	0.035	W	0.0032	0.0028	0.0094	0.011
S	0.1488	0.0852	0.1496	−0.0461	Re	0.0096	0.0068	0.0117	
K	−0.4969	−0.2329	−0.2276		Os	0.0133	0.0099	0.0127	
Ca	−0.2508	−0.1668	−0.1753	−110.0	Ir	0.0164	0.0094	0.0121	
Sc	−0.1013	−0.0477	−0.1258		Pt	0.0179	0.0086	0.0056	0.0089
Ti	−0.0532	−0.0174	−0.0820	−0.18	Au	0.0018	0.0137	−0.0039	0.0028
V	−0.0367	−0.0174	−0.0463	−0.019	Hg	−0.0153	0.0011	−0.0091	
Cr	−0.0156	−0.0089	−0.0173	−0.0103	Tl	−0.0286	−0.0174	−0.0068	
Mn	−0.0395	−0.0221	−0.0393	−0.026	Pb	−0.0284	−0.0182	−0.0051	−0.046
Co	0.0042	0.0029	0.0036	0.0026	Bi	−0.0254	−0.0154	−0.0042	
Ni	0.0087	0.0061	0.0071	0	Po	−0.0302	−0.0194	−0.0443	
Cu	−0.0329	−0.0238	−0.0214	−0.0084	La	−0.0491	−0.0270	−0.0320	−18.3
Zn	−0.0130	−0.0040	−0.0095		Ce	−0.0346	−0.0176	−0.0309	−9.10
Ga	−0.0009	0.0119	−0.0031		Pr	−0.0316	−0.0158	−0.0301	
Ge	0.0181	0.0133	0.0142	0.014	Nd	−0.0307	−0.0156	−0.0290	−0.76
As	0.0313	0.0241	0.0431	0.0041	Pm	−0.0271	−0.0122	−0.0279	
Se	0.0423	0.0274	0.0456		Sm	−0.0269	−0.0130	−0.0497	
Rb	−0.4041	−0.1789	−0.1711		Eu	−0.0800	−0.0500	−0.0265	
Sr	−0.1478	−0.1015	−0.0872		Gd	−0.0255	−0.0128	−0.0254	
Y	−0.0657	−0.0368	−0.0674	−0.275	Tb	−0.0231	−0.0109	−0.0248	
Zr	−0.0234	−0.0157	−0.0521	−0.210	Dy	−0.0226	−0.0107	−0.0244	
Nb	−0.0112	0.0005	−0.0259	−0.013	Ho	−0.0229	−0.0113	−0.0233	
Mo	−0.0024	−0.0005	−0.0041	0.0027	Er	−0.0200	−0.0089	−0.0230	
Tc	0.0155	0.0109	0.0131		Tm	−0.0198	−0.0089	−0.0400	
Ru	0.0201	0.0142	0.0166		Yb	−0.0600	−0.04	−0.0215	
Rh	0.0152	0.0116	0.0111		Lu	−0.0168	−0.0066	−0.0192	

## References

[1] Mintz B. (1999). The influence of composition on the hot ductility of steels and to the problem of transverse cracking. ISIJ Int..

[2] Ryan M.P., Williams D.E., Chater R.J., Hutton B.M., McPhail D.S. (2002). Why stainless steel corrodes. Nature.

[3] Costa e Silva A. (2006). Thermodynamic aspects of inclusion engineering in steels. Rare Met..

[4] Gollapalli V., Rao M.B.V., Karamched P.S., Borra C.R., Roy G.G., Srirangam P. (2018). Modification of oxide inclusions in calcium-treated Al-killed high sulphur steels. Ironmak. Steelmak..

[5] Wagner C. (1952). Thermodynamics of Alloys.

[6] Lupis C.H.P., Elliott J.F. (1966). Generalized interaction coefficients. Acta Metall..

[7] Darken L. (1967). Thermodynamics of binary metallic solutions. Trans. Met. Soc. AIME.

[8] Pelton A.D., Bale C.W. (1986). A modified interaction parameter formalism for non-dilute solutions. Metall. Trans. A.

[9] Bale C.W., Pelton A.D. (1990). The unified interaction parameter formalism: Thermodynamic consistency and applications. Metall. Trans. A.

[10] Costa e Silva A. (2016). Interaction parameters of oxygen and deoxidants in liquid iron. J. Min. Metall. Sect. B Metall..

[11] Waseda Y. (2012). Interaction parameters in metallic solutions estimated from liquid structure and the heat of solution at infinite dilution. High Temp. Mater. Process..

[12] Ding X.Y., Fan P., Wang W.Z. (1999). Thermodynamic calculation for alloy systems. Metall. Mater. Trans. B.

[13] Ding X., Fan P., Han Q. (1994). Models of activity and activity interaction parameter in ternary metallic melt. Acta Metall. Sin..

[14] Ueno S., Waseda Y., Jacob K.T., Tamaki S. (1988). Theoretical treatment of interaction parameters in multicomponent metallic solutions. Process Metall..

[15] Fan P., Chou K.C. (1999). A self-consistent model for predicting interaction parameters in multicomponent alloys. Metall. Mater. Trans. A.

[16] Wang F.M., Li X.P., Han Q.Y., Zhang N.X. (1997). A model for calculating interaction coefficients between elements in liquid and iron-base alloy. Metall. Mater. Trans. B.

[17] Zhang N., Chen W., Chen X., Ding X., Zhou G. (2013). Modeling Activity and Interaction Coefficients of Components of Multicomponent Alloy Melts: An Example of Iron Melt. High Temp. Mater. Process..

[18] Ding X., Fan P., Luo L. (1998). Alloy Melts Thermodynamic Model: Prediction and Software Development.

[19] Neuhausen J., Eichler B. (2003). Extension of Mediema’s Macroscopic Atom Model to the Elements of Group 16 (O, S, Se, Te, Po).

[20] Hino M., Ito K. (2010). Thermodynamic Data for Steelmaking.

[21] Inoue R., Suito H. (1994). Calcium desulfurization equilibrium in liquid iron. Steel Res..

[22] Hillert M. (1980). Empirical methods of predicting and representing thermodynamic properties of ternary solution phases. Calphad.

[23] Tanaka T., Morita Z.-I., Gokcen N.A., Iida T. (1993). Thermodynamic relationship between enthalpy of mixing and excess entropy in liquid binary alloys. Zeitschrift für Metallkunde.

[24] Miedema A., De Chatel P., De Boer F. (1980). Cohesion in alloys—Fundamentals of a semi-empirical model. Physica B+C.

[25] Niessen A., Miedema A., De Boer F., Boom R. (1988). Enthalpies of formation of liquid and solid binary alloys based on 3d metals: IV. Alloys of cobalt. Physica B+C.

[26] Niessen A.K., de Boer F.R., Boom R., De Châtel P.F., Mattens W.C.M., Miedema A.R. (1983). Model predictions for the enthalpy of formation of transition metal alloys II. Calphad.

[27] Wu X.S., Yan X.H., Ma B.K., Lin Z.J., Yang X.Z. (1995). Calculation of the Heat of Formation of Ternary Compounds—P-Ga-as, N-Ga-as, N-Ga-P. Acta Phys. Sin..

[28] Taguchi K., Ono-Nakazato H., Nakai D., Usui T., Marukawa K. (2003). Deoxidation and desulfurization equilibria of liquid iron by calcium. ISIJ Int..

[29] Wu Y., Wang L., Du T. (1985). Thermodynamics of rare earth elements in liquid iron. J. Less-Common Met..

[30] Zhang R.F., Liu B.X. (2002). Proposed model for calculating the standard formation enthalpy of binary transition-metal systems. Appl. Phys. Lett..

[31] Sun S.P., Yi D.Q., Jiang Y., Zang B., Xu C.H., Li Y. (2011). An improved atomic size factor used in Miedema’s model for binary transition metal systems. Chem. Phys. Lett..

[32] Chen X.-Q., Podloucky R. (2006). Miedema’s model revisited: The parameter for Ti, Zr, and Hf. Calphad.

[33] Chartrand P., Pelton A.D. (2000). On the choice of “Geometric” thermodynamic models. J. Phase Equilib..

